# Social and nutritional factors shape larval aggregation, foraging, and body mass in a polyphagous fly

**DOI:** 10.1038/s41598-018-32930-0

**Published:** 2018-10-03

**Authors:** Juliano Morimoto, Binh Nguyen, Shabnam Tarahi Tabrizi, Fleur Ponton, Phillip Taylor

**Affiliations:** 1 Department of Biological Sciences, Macquarie University, North Ryde, NSW 2109 Australia; 2 Programa de Pós-Graduação em Ecologia e Conservação, Federal University of Paraná, 19031, CEP: 81531-990 Curitiba, Brazil

## Abstract

The majority of insect species have a clearly defined larval stage during development. Larval nutrition is crucial for individuals’ growth and development, and larval foraging success often depends on both resource availability and competition for those resources. To date, however, little is known about how these factors interact to shape larval development and behaviour. Here we manipulated the density of larvae of the polyphagous fruit fly pest *Bactrocera tryoni* (‘Queensland fruit fly’), and the diet concentration of patches in a foraging arena to address this gap. Using advanced statistical methods of machine learning and linear regression models, we showed that high larval density results in overall high larval aggregation across all diets except in extreme diet dilutions. Larval aggregation was positively associated with larval body mass across all diet concentrations except in extreme diet dilutions where this relationship was reversed. Over time, larvae in low-density arenas also tended to aggregate while those in high-density arenas tended to disperse, an effect that was observed for all diet concentrations. Furthermore, larvae in high-density arenas displayed significant avoidance of low concentration diets – a behaviour that was not observed amongst larvae in low-density arenas. Thus, aggregation can help, rather than hinder, larval growth in high-density environments, and larvae may be better able to explore available nutrition when at high-density than when at low-density.

## Introduction

In holometabolous insects, larval foraging behaviour largely determines individual fitness (Chapman, 1998). Poor developmental conditions marked by low resource availability – such as when food is scarce and there is high larval competition – often affects both larval developmental time and body size in adulthood [e.g.^1–10^]. Adult body size tends to correlate positively with female fecundity as well as male mating performance and reproductive success^5,11^; accordingly, larval foraging behaviour is under productivity selection in females and sexual selection in males^11–15^, with profound effects on behavioural and evolutionary processes such as cognitive task performance, survival, reproduction, and ultimately sexual selection and sexual conflict^6,16–18^.

The quantity of resources in a food patch and the number of competing foragers are important determinants of larval responses to developmental conditions^19^. To maximize resource acquisition for investment in fitness traits of adulthood^20,21^, larvae are expected to avoid competition with conspecifics, and to prefer patches of highest resource availability. The rationale for this is simple; if the resources are poor or the number of individuals sharing a finite resource is high, the benefits of foraging on that patch may be outweighed by the potential benefits of leaving that patch to seek resources elsewhere. Thus, the ideal situation may be that in which larvae forage in resource-rich food patches without competition. Research across insect taxa has shown that insect larvae have well-defined optimum diets that sustain development and growth, and produce high quality adults^22–27^, that an excess of nutrients can be detrimental and even compensated for when larvae have a choice to select their food [e.g.^28–31^]. For social interactions, however, the rule is far less intuitive. Larval aggregations are common in many insects^32,33^. Although such social interactions may increase foraging competition, larval aggregations can confer physiological and behavioural benefits that sustain larval growth and development^34–45^. As a result, larvae may maximize development in a high-quality diet with some degree of social interactions and aggregation, provided that competition is not so high that the benefits of aggregation are negated. For instance, *Drosophila* larvae can benefit from occupying patches that are shared with conspecifics, although the increase in competition can in some cases offset the benefits of social behaviour^45^ [see also^46–48^]. This hypothesis is derived from the premise that social and nutritional factors interact to shape larval behaviour and growth during development. To date, however, there have been very few direct empirical tests of this hypothesis.

An early attempt to demonstrate interactions between nutritional and social factors as determinants of larval development showed that, in the gregarious caterpillar *Hemileuca lucina*, social environment interacts with the quality of the food source to determine larval growth at mild temperatures^37^. This investigation only contrasted caterpillars in solitary and groups of a fixed size (10 individuals), and only investigated development on two related-food sources, young vs. mature leaves of *Spiraea latifolia*. Although providing a useful demonstration of concept, this dichotomous approach – i.e. solitary *vs* groups, young *vs* mature leaves – has limited scope for understanding the interaction between social and nutritional factors driving the ecology of larval development. Other studies have shown the importance of larval aggregation in feeding and growth rates, insect-plant interactions, larval defence against predators, and larval thermoregulation [e.g.^34–44^]. However, there has been no detailed investigation of how the social and nutritional environments of larvae interact to shape development and performance. Key questions remain unanswered though, as to ‘how does the number of foraging larvae influence larval aggregation?’; ‘When resource availability decreases, do larvae aggregate to the same extent as to when resources are abundant?’; and ‘What are the implications of density- and diet-dependent larval aggregation to larval growth and foraging behaviour?’

In the present study, we addressed these key questions of the interaction between nutritional and social factors driving larval foraging decisions and performance in the tephritid fruit fly *Bactrocera tryoni* (aka ‘Queensland fruit fly’ or ‘Qfly’). Some tephrtids are highly polyphagous and are amongst the most damaging insect pests of horticulture globally^49–51^. *Bactrocera tryoni* is able to infest more than 150 different fruits^49,52^; the wide diversity of fruit that are exploited by *B. tryoni*, and variability of nutrients available in infested fruit, make this species well suited for investigation of larval nutritional ecology. Here we first designed circular foraging arenas containing patches of varying macronutrient concentration, where different densities of larvae were allowed to forage. Larvae foraged freely in choice and no-choice arenas, which allowed us to investigate the diet- and density-dependent effects of larval developmental environment on foraging behaviour and larvae body mass. Using statistical methods of machine learning and linear regression, we tested whether tendency to aggregate and size of aggregations depended on the larval density and diet, by allowing groups of several larval densities to forage in arenas of varying diet concentration within which each arena contained multiple patches of the same diet. We then tested how larval density and aggregation affected larval body mass across different diets. Finally, we investigated how larval density influenced larval foraging decisions when facing choices amongst patches with varying resource availability.

## Predictions

1. Previous studies in other species have shown that larvae prefer to occupy patches that are shared with conspecifics [e.g.^45^]. Thus, we predicted that an increase in larval density should increase aggregation formation as well as aggregation size amongst diet patches. However, this effect could be diet-dependent, whereby macronutrient-poor diets could support smaller aggregations whereas macronutrient-rich diets would support larger aggregations. As a result, we predicted that aggregates should be smaller in macronutrient-poor diets than in macronutrient-rich diets.
2. In other insects, larval aggregation can facilitate feeding [e.g.^40^]. We therefore predicted that treatments with high larval aggregations should have larvae with higher body mass. However, macronutrient-poor diet is known to reduce larval body mass (see ‘Introduction’). As a result, we predicted that larval body mass should be lower in macronutrient-poor diets compared with macronutrient-rich diets.

## Materials and Methods

### Fly stock and egg collection

We collected eggs from a laboratory-adapted stock of *B. tryoni* (>17 generations-old). The colony has been maintained in non-overlapping generations in a controlled environment room (humidity 65 ± 5%, temperature 25 ± 0.5 °C) with light cycle of 12 h light: 0.5 h dusk:11 h dark: 0.5 h dawn). Adults were provided a free-choice diet of hydrolysed yeast (MP Biomedicals, Cat. n° 02103304) and commercial refined sucrose (CSR® White Sugar), while larvae were maintained using the Chang-2006 gel-based diet formulation of Moadeli, *et al*.^53^ for the last 7 generations (previously maintained on a carrot-based diet). We collected the eggs in a 300 mL semi-transparent white plastic bottle that had numerous perforations of <1 mm diameter through which females could insert their ovipositor and deposit eggs. The bottle contained 20 mL of water, to maintain high humidity. Eggs were collected for 2 h, and were then transferred to Chang-2006 gel-based larval diet with a soft brush, where eggs were allowed to hatch and larvae to develop for four days, until they reached 2^nd^ instar stage.

### Experimental diets and foraging arena

We used 5 experimental diets that varied in macronutrient (i.e., yeast for protein and sugar for carbohydrate) concentration: our control and reference 100% Chang-2006 gel-based diet, which has proven effective for the larvae of this species^53^, followed by diets with 80%, 60%, 40%, and 20% macronutrient concentration relative to the control diet (see Supplementary Tables for recipes). 20 mL of diet was poured into 90 mm diameter Petri dishes and allowed to set. We also prepared an agar solution that contained the same components as the gel diets except that no yeast or sugar was included. 20 mL of the agar solution was used to cover 90 mm diameter Petri dishes that then served as “foraging arenas”. After setting, five equally spaced holes were made in the agar base of each foraging arena by perforating it with a 25 mm diameter plastic tube. The same tube was used to cut discs from the experimental diets. The discs of experimental diets were then deposited – in order or randomly – in the holes that had been cut in the agar base of the foraging arenas (see Fig. S1). Because the agar solution did not contain macronutrients, we considered the remaining areas of agar base as ‘no choice’ foraging option. Thus, larvae had a total of 6 options (i.e., 5 experimental diets + agar base). The pH of all experimental diets and the agar base was adjusted to 3.8–4 with citric acid. For the experiment, hydrolyzed yeast and sucrose were obtained from MP Biomedicals (Cat. n° 02103304 and 02902978, respectively), Brewer’s yeast was obtained from Lallemand (Cat n° LBI2250), Nipagin was obtained from Southern Biological (Cat n° MC11.2), and all other chemicals composing the diet (e.g., citric acid [see^53^]) were obtained from Sigma Aldrich®.

### Experimental procedures and statistical analyses

For all experiments, we placed 2^nd^ instar larvae at the centre of the foraging arena (see Fig. S1) that was then covered with the lid to minimize the loss of moisture. To minimize potential for effects of visual cues on larval diet choices, the foraging arenas were placed in a dark room. Foraging arenas were set up at 4 larval densities: 10, 25, 50, and 100 larvae. All larvae were released in the arena simultaneously. Larvae were not starved before the onset of the experiments. We did not observe cannibalism or escapes (larval counts were the same at the beginning and at the end of the experiments). All statistical analyses were performed using R version 3.4.0 and plots were performed using the package ‘ggplot2’^54,55^.

#### Experiment 1: Larval aggregation

To test effects of density and diet on larval aggregation and growth, for all diets and across all larval densities, we set up foraging arenas that contained 5 equidistant food patches (distance of ca. 35 mm from the centre of each patch) of the same diet concentration (e.g., all patches with 100% diets) (see Fig. S1). We then numbered the patches, and assessed the number of larvae in each of the diet patches as well as on the agar base at 1 h, 2 h, 4 h, 6 h, 8 h, and 24 h after larvae were placed in the arena. We observed that larvae could move across the diameter of the foraging arena in less than 1 min, meaning that the time points used in the experiment were ample to allow larvae to explore the entire foraging arena. Four replicates were set up per larval density per diet (*N* = 80 foraging arenas). After 24 h, 3 larvae per diet per larval density per replicate were selected from each foraging arena and weighed on a ME5 Sartorius® scale (0.001 g precision) to obtain an estimate of average larval body mass. We tested the effects of larval density, diets, and their interaction, using two-way ANOVA model that included replicate as a covariate. To measure larval aggregation, we calculated an ‘aggregation index’ (*AI*) which was the sum of the absolute residuals of our observed data against the machine learning random predictions of a density-dependent random distribution; the procedure to obtain *AI* was as following:

1. We simulated the choices of larvae in foraging arenas with density 10, 25, 50, 100, and 200 larvae choosing amongst 6 patches, where the larvae were equally likely to display choice for any of the options (i.e., the choices for each patch were displayed with equal probability *p*_*n*_ = 1/6, where *p*_*n*_ is the probability of a larvae choosing a given patch). We extrapolated our simulation for larval densities of 10, 25, 50, 100, and 200 larvae in order to build a robust function of density-dependent aggregation (see Fig. S2).
2. We then obtained the residual distribution of our empirical data and the simulated density-dependent model against the exact random distribution, calculated simply by dividing the larval density by the number of patch options (i.e. *δ*/6, where *δ* = larval density); We then fitted a random forest machine learning regression using the package ‘randomForest’^56^ to obtain a model that predicted the behaviour of the residuals. The random forest regression was cross-validated using the package ‘rfUtilities’^57^ (Fitted Mean Square Error of the model: 0.009; Median Cross-validation RMSE: 0.036); To build the model, 80% of the simulated data was used in the training phase while 20% was used in the test phase. The model performed accurately during the test phase (Mean Square Error in the Test dataset: 0.038).
3. Next, we used the machine learning model to predict the expected distribution of residuals in our dataset using the ‘predict’ function, and calculated the aggregation index *(AI)* as the difference between the observed sum of residuals and the predicted sum of the residuals obtained with the machine learning regression algorithm.

The machine learning model provides more accurate predictions of the expected distribution of the residuals than conventional linear model. For instance, the MSE (mean square error) of the machine learning model in the test data set was 0.00404 whereas the MSE estimated using conventional linear model was 0.0107, suggesting that the machine learning model was ~2.7 times more accurate in its prediction. We therefore opted to use the machine learning approach to account for non-linear behaviour of the residuals as the density of larvae in the foraging arenas increases (see Fig. S2). When we modelled *AI* using general linear model followed by a two-way ANOVA to determine the effect of time, larval density, diet, and their two-way interactions, we transformed *AI* (i.e. *AI*^2.25^) in order to stabilize the variance across larval densities (Levene’s test: F_3,476_ = 0.560, p = 0.641) and diets (Levene’s test: F_4,475_ = 0.548, p = 0.700). To test for the effects of aggregation on larval body mass, we used an ANOVA with the average aggregation index over time, larval density, and diet, as well as the two-way interactions between these factors. For statistical inference, we transformed larval body mass (i.e., *Larval mass*^0.3^) for homogeneity of variances across larval densities (Levene’s test: F_3,76_ = 0.591, p = 0.622). To calculate the average size of the largest aggregation, we sampled the aggregation with the highest larval count, and calculated the proportion of individuals of the group that were found in that aggregation (*ρ*) as *ρ* = *α*/*δ*, where *α* = the number of larvae in the largest patch and *δ* = the larval density of the group. To test for the effects of time, larval density, diet, and their two-way interactions we used a generalized linear model (GLM) with *Binomial* distribution – as we were dealing with proportion data – and *quasi* extension, to account for overdispersion of the data. Plots are of the raw data.

#### Experiment 2: Larval foraging

For larval foraging assays, the foraging arena contained one patch of each experimental diet, and we assessed the number of larvae selecting each diet across all larval densities (see above) at 1 h, 2 h, 4 h, 6 h, and 8 h after larvae were placed in the arena. Foraging arenas contained equidistant food patches (i.e. 100%, 80%, 60%, 40% and 20% nutrient concentration) in different orders within the arena (see Fig. S1); we controlled for the order of the patches in all models, which had no effect in the results (see ESM). We fitted a multinomial logistic regression model using the ‘multinom’ function of the “nnet” package^58^. To test for foraging propensity (i.e., the likelihood of the larvae foraging as opposed to staying in the agar base), we controlled for the order of the food patches while investigating the main effects of time, larval density, and their interaction. Agar base (no choice) was the reference level. To test for dietary choices (i.e., the likelihood of the foraging larvae choosing one patch or another), we used the same multinomial logistic regression, but this time only considering those larvae that chose to forage. By using the standard diet (100% macronutrient concentration) as our reference level, we could then infer the relative dietary preferences of larvae that foraged. Statistical inferences for multinomial logistic regressions were made based on 95% and 99% confidence intervals for each larval density separately.

## Results

### Experiment 1: High larval density increases larval body mass

**Figure 1:**
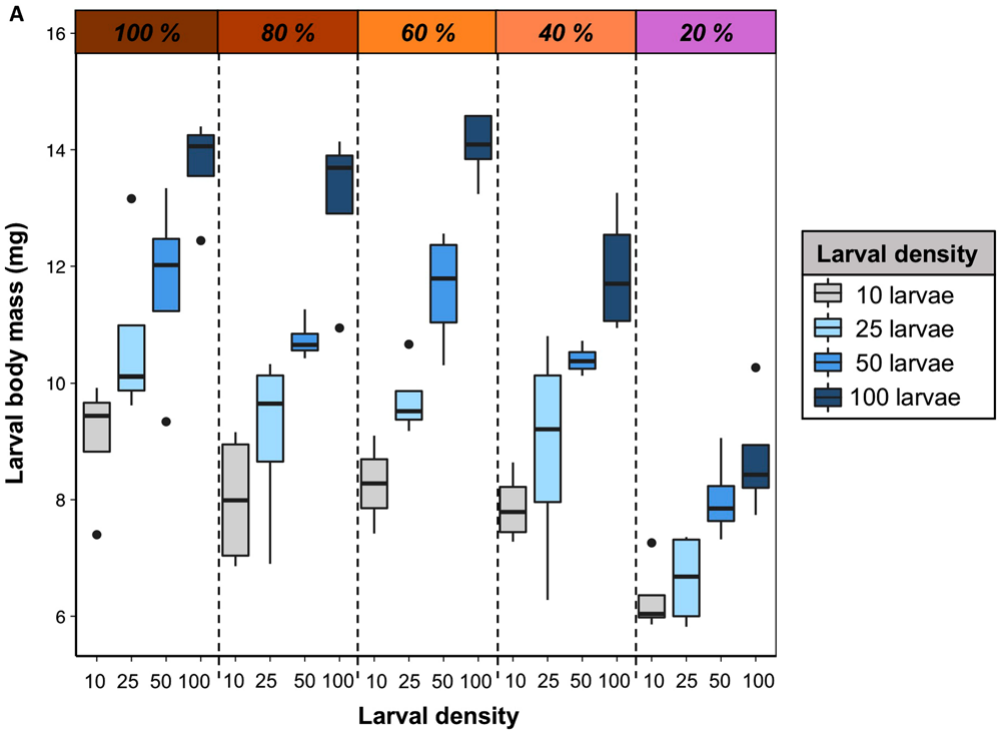
Larval density increases larval body mass across diet dilutions. Body mass (mg) of larvae from different larval densities and from across diets, at the end of our experiment (24 h, see Methods for details).

We first tested the influence of larval density on growth. Our results showed highly significant positive effects of diet concentration and larval density on body mass (Table S1), although there was no significant interaction between these factors. Body mass increased steadily with larval density in the foraging arena and consistently across all diets (Fig. 1). However, diet concentration also affected larval body mass, as larvae from foraging arenas with diluted diets (i.e. 40% and 20% macronutrient concentration) had lower body mass than larvae from arenas containing more concentrated diets (Fig. 1).

### Experiment 1: Larval density affects larval aggregation in a diet-dependent manner

**Figure 2:**
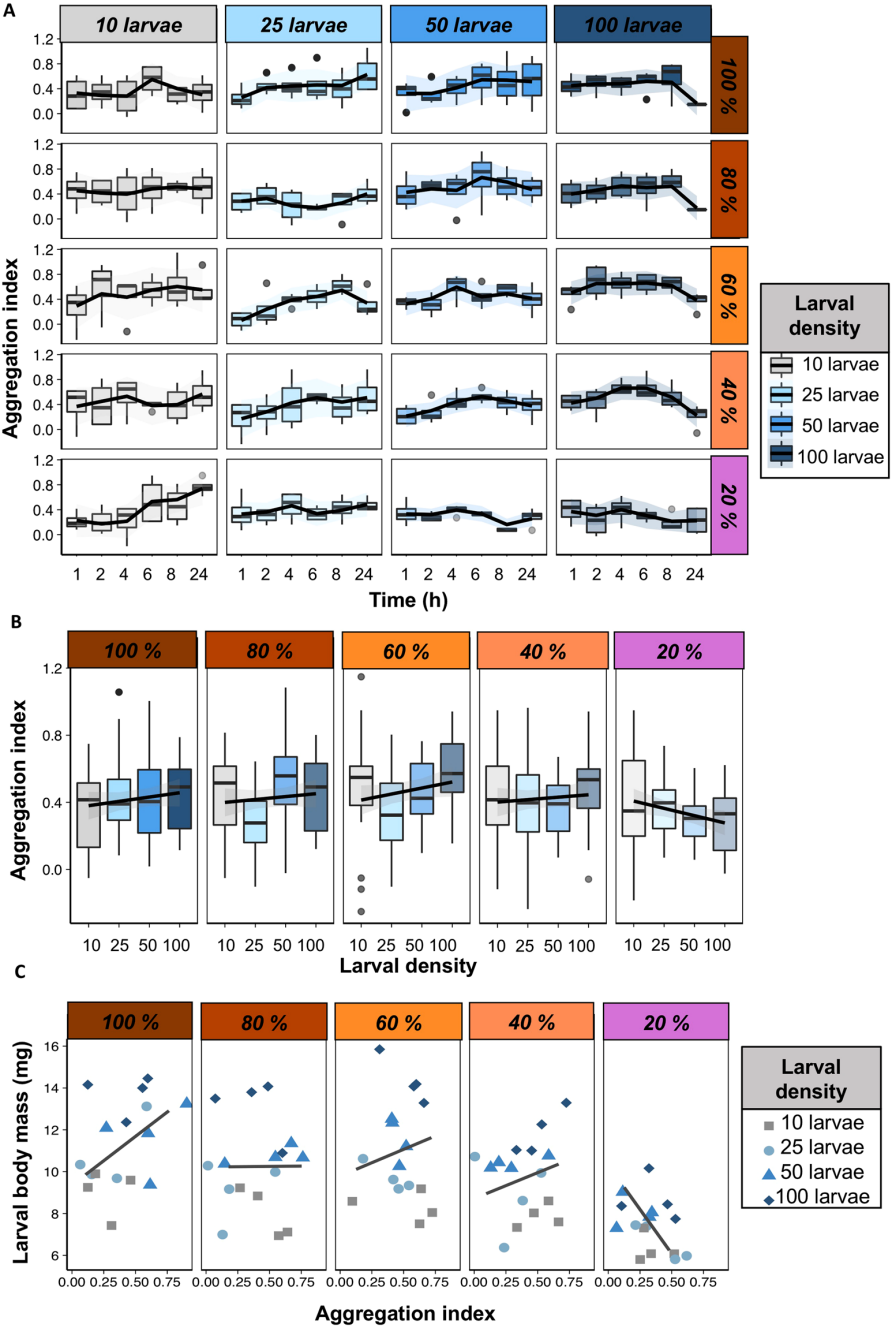
The relationship between body mass and aggregation. (**A**) Larval aggregation index (*y-*axis) over time (*x-*axis) across larval densities (horizontally) and across diets (vertically). Lines were drawn using the ‘loess’ method in the package ‘ggplot2’ in R, and indicate the trend in the data. (**B**) Average larval aggregation index (*y*-axis) on larval density (*x*-axis) over all time points in our experiment. Lines were drawn using the ‘lm’ method in the package ‘ggplot2’ in R, and indicate the trend in the data. (**C**) The relationship between larval body mass and the average aggregation index. Colours and shapes indicate the larval density. Lines were drawn using the ‘loess’ method in the package ‘ggplot2’ in R, and indicate the trend in the data.

We investigated whether larval density modulated larval aggregation, and whether this relationship was affected by diet concentration. We found significant interaction between effects of diet concentration and larval density on the aggregation index (Table S2), whereby larvae in high-density arenas aggregated more in high macronutrient concentration diets (>40%) and less in low macronutrient concentration diets (20%, Fig. 2A,B).

There was a significant interaction between time and larval density, whereby larvae in low-density arenas (10 larvae) increased aggregation as time foraging passed, while the opposite pattern was observed for high-density arenas (100 larvae) (Table S2, Fig. 2A,B). This was particularly evident for low-density arenas with low macronutrient concentration diets (see Fig. 2A). This is important because if the larvae were simply coalescing in the same location (i.e., not seeking to aggregate but converging to the same location with high quality food substrate), we would expect larvae in low-density arenas to show the same pattern for high- and low diet concentration. Instead, the results show the opposite is true, whereby larvae in low-density arenas tended to aggregate more over time with low diet concentration than with high diet concentration (Fig. 2A). This provides evidence that larvae seek to aggregate, especially when foraging in low-density arena and with low-resource food substrates. Arenas with density of 25 and 50 larvae showed the same trend as arenas with 10 and 100 larvae, respectively, although with lower magnitude (Fig. 2A,B).

### Experiment 1: The relationship between larval aggregation and larval body mass is diet-dependent

Next, we tested the relationship between larval aggregation and body mass. We found that aggregation had an overall highly significant positive effect on larval body mass when diet concentration was 40% or greater but that a negative trend was instead observed when diet concentration was 20% (Fig. 2C, Table S3). There was a significant effect of diet concentration and larval density, but there were no significant interactions between larval density and diet concentration, larval density and aggregation index, nor between diet concentration and aggregation index (Table S3). These results provide evidence for a positive relationship between larval aggregation and larval body mass, and revealed that in some cases nutrient concentration in the diet can be a strong modulator of this relationship.

### Experiment 1: Larval density and diet influence the size of larval aggregations

**Figure 3:**
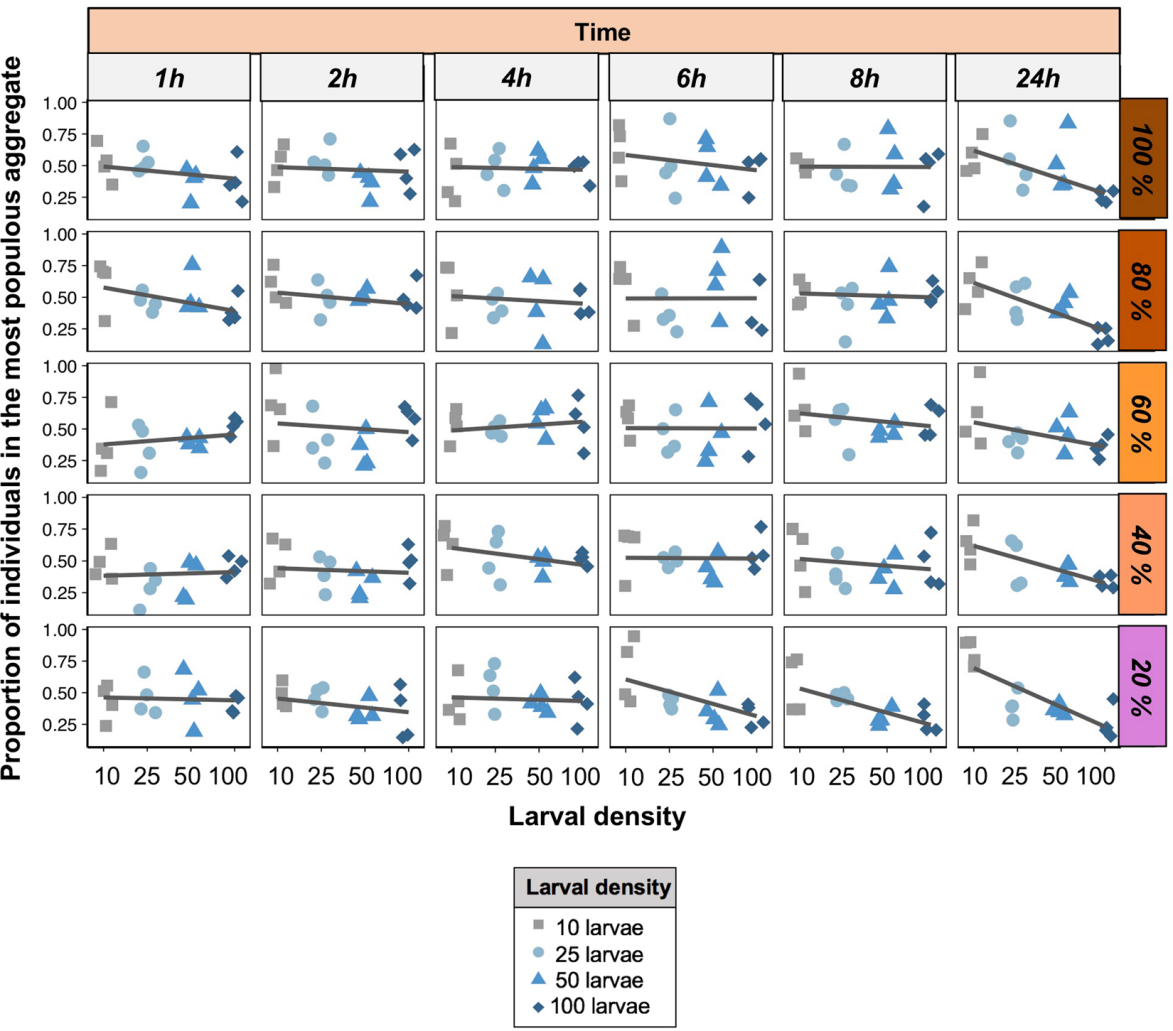
Proportion of larvae in aggregates. The proportion of individuals in the most populous aggregate over time (horizontally) across diets (vertically). Shapes and colours indicate larval density. Lines were drawn using the ‘lm’ method in the package ‘ggplot2’ in R, and indicate the trend in the data.

Previous studies have shown that larval aggregation can help larvae to feed more efficiently, potentially leading to an increase in larval body mass (see for instance)^40,59^. If this is true, an aggregation could become a ‘hotspot’ for other larvae, and we would expect that arenas with high larval densities would have few large aggregations. This could explain the relationships between larval aggregation and body mass and also the relationship between larval density and larval aggregation. Alternatively, high larval density could make larvae more inclined to disperse in order to minimize competition and, as a result, form smaller aggregations at more locations, hence exploiting a greater number of food patches. Our results showed a significant interaction between the effects of larval density and time, and larval density and diet concentration on the proportion of individuals in the largest aggregation (see Table S4, Fig. 3). These results demonstrate that (i) arenas containing diluted diets (i.e., 20% and 40%) had relatively more larvae in the most populous aggregations than did arenas containing more concentrated diets, (ii) low larval density arenas (i.e., 10 larvae) had aggregations that contained relatively more larvae compared with higher density arenas (i.e., 25, 50, 100), (iii) high density arenas (i.e., 100 larvae) were more evenly distributed compared with low density arenas, whereas the opposite effect was found for low density arenas (i.e., 10 larvae), and iv) the proportion of larvae in the most populous aggregation decreased in diluted diets in high density arenas, an effect that was not observed for low-density arenas (see Fig. 3). Importantly, in arenas with 20% concentration, there was a sharp trend for the proportion of individuals in the most populous aggregate to decrease as larval density in the group increased 6 h after the onset of the experiment, which was maintained until the end (Fig. 3). This pattern was not observed in other diets where the decreasing relationship between larval density and the proportion of individuals in the most populous aggregate was evident only 24 h after the onset of the experiment (see Fig. 3). These findings support the hypothesis that high larval density promotes larval movement, whereby larvae formed smaller aggregations that exploit patches more evenly.

### Experiment 2: Larval density shapes larval foraging behaviour

**Figure 4:**
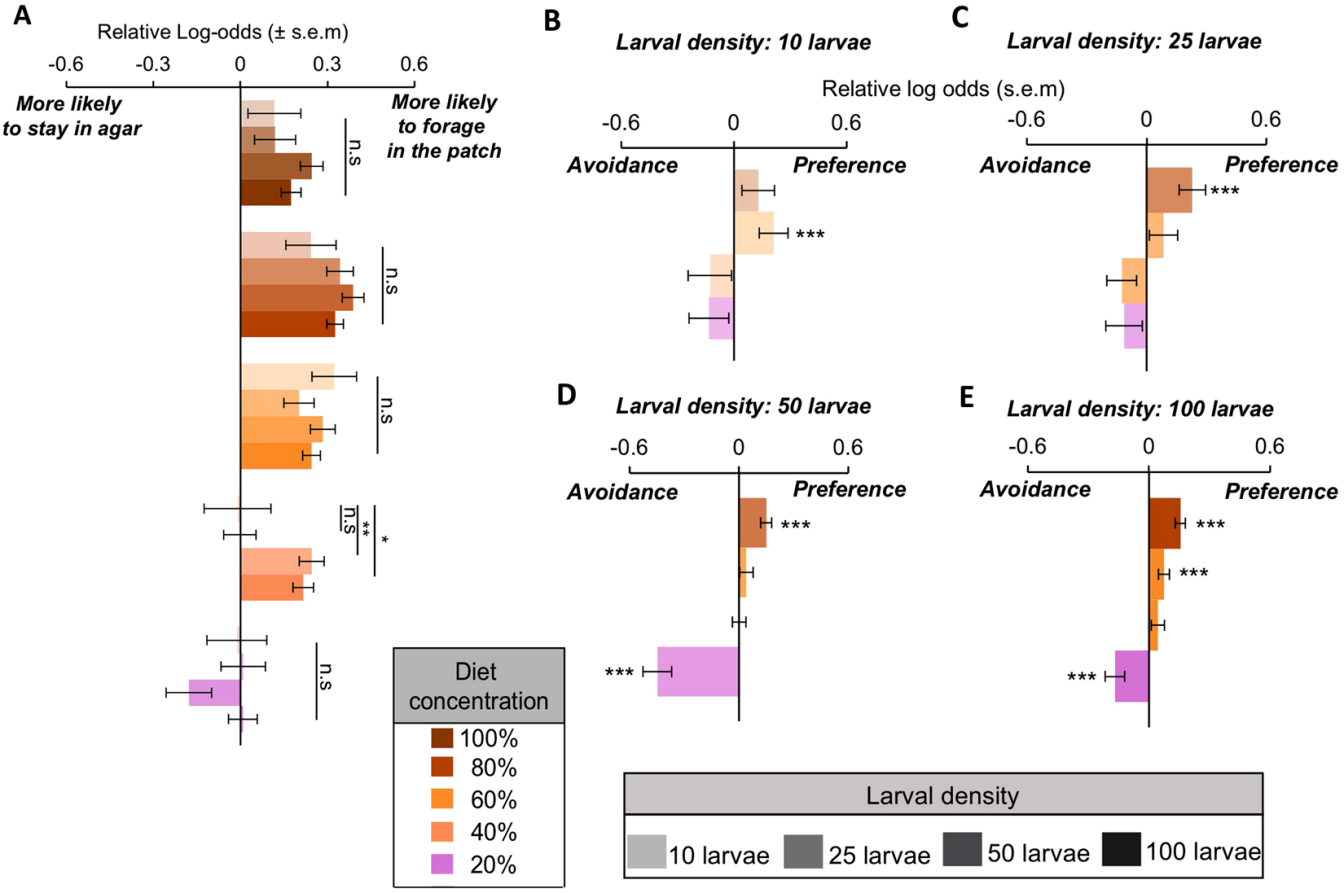
Larval foraging propensity. (**A**) Relative log-odds of larvae making a choice to forage in a given food patch relative to staying in agar (no choice). Shades represent different larval densities: 10, 25, 50, and 100 larvae. p-values obtained using Students’ *t*-distribution. Note that relative log-odds are calculated using the control 100% diet as reference. Log-odds > 0: more likely to choose a given patch relative to staying in agar, Log-odds < 0, less likely to choose a given patch relative to staying in agar. s.e.m = standard error of the mean. (**B**–**E**) Relative log-odds of larvae patch preferences. Patch with standard diet (100% macronutrient concentration) was used as the reference level. ***Non-overlapping 99% confidence intervals. s.e.m = standard error of the mean.

Next, we measured how larval density influenced larvae foraging propensity, as well as larvae foraging decisions when larvae have a choice amongst patches with varying diet concentrations. By using a multinomial logistic regression model that used ‘no choice’ (i.e. agar base) as our reference level, we could assess larval foraging propensity over time. Our results showed that larvae were more likely to forage in any given patch than to not forage at all, and the propensity of foraging was particularly high for patches of high nutrient concentration independent of larval density (Fig. 4A, Table S5, Fig. S3). Interestingly, the range of diets in which larvae foraged was greater for arenas containing 50 and 100 larvae and included the patch with 40% diet in addition to the 100%, 80% 60% patches that were more dominant for arenas of lower larval density (Fig. 4A). These findings show that larvae are generally more prone to forage in high-quality patches, and that larval foraging propensity is density-independent.

We then tested whether larval density affected larval diet choices, using again a multinomial logistic regression although this time we used the standard diet (i.e. 100%) as our reference diet. Here, we excluded non-foraging larvae, and modelled the behaviour of larvae that were actively foraging in one of the food patches in the previous experiment. In arenas with low larval density (10 larvae), larvae displayed a significant preference for diets with 60% macronutrient concentration relative to the standard (100%) diet (Fig. 4A, Table S6). However, as larval density increased (25 and 50 larvae), there was a shift in preference toward the patch containing 80% macronutrient concentration (Fig. 4C,D), and finally, when larval density was the highest (100 larvae), larvae displayed statistically significant preferences for both 60% and 80% macronutrient patches compared to the standard diet (Fig. 4E). More importantly, though, is that only larvae in arenas with high density (50 and 100 larvae) displayed significant avoidance of low concentration patches of 20% macronutrient concentration (Fig. 4D,E).

## Discussion

In this study, we demonstrate how key ecological factors interact to determine larval foraging behaviour and growth in *B. tryoni*. Our findings showed that larval aggregation increased with larval density in a diet-dependent manner, and promoted larval body mass across all diets. Importantly, larval density modulated the size of larval aggregations, and influenced larval foraging behaviour when larvae experienced patches with varying concentrations, highlighting a role of social interactions and population density for larval behaviour. Our findings provide insight into larval foraging decisions of fruit flies, and more generally, provide insight into broad ecological patterns arising from nutrition and intraspecific competition within groups and populations. Fruit fly larvae are commonly found in aggregations within a fruit^9,10,60^. Furthermore, fruits can be heterogeneous foraging environments for larvae [e.g.^61^], and the nutritional composition of fruit can change as larvae develop [see^62–64^]. Therefore, the density of larvae and local diet quality might determine larval movement within a fruit in search of more nutritious and less competitive foraging sites. It is important to note that it is unlikely that our findings apply to movement of larvae between fruits. Crawling out of fruits is dangerous owing to risks of predation^65^ [reviewed by^66^] and desiccation. In nature, *B. tryoni* females modulate their oviposition behaviour to minimize intra-specific competition amongst larvae^67^, and it is reasonable to expect the larvae to very rarely move between fruits.

High population density can force animals to change their behaviour and expand their niche due to inter- and intra-specific competition, and this is a well-established ecological principle observed in both the laboratory and in nature^68,69^. Even though larvae are prone to aggregate, an increase in larval density could increase larval competition within large aggregations, which could in turn drive larvae to disperse and form smaller aggregations across different locations. The smaller aggregation size observed in high-density arenas support this idea, meaning that larval aggregations formed in high-density arenas were proportionally smaller than those formed at lower densities. Moreover, larval aggregations were proportionally smaller as the density increased and the larvae spent more time foraging, suggesting that social interactions within larger aggregations are likely to induce more frequent movement by the larvae. As the larvae move more often, they are more likely to find new (and unexplored) food patches, and are therefore more likely to explore patches more evenly. The influence of larval density on larval aggregation and growth could therefore be a plastic response to intraspecific competition because it could lead to better larval foraging decisions and a broader niche exploration^45,70^. The findings that high larval density also influence larval foraging behaviour in ways that decrease larval foraging propensity on resource-poor diet patches provide further support for the idea that high larval density promotes exploration of the foraging environment and effective exploitation of nutritional resources. Individuals of many species use social cues when making decisions^71^, and recent models have predicted that social interactions could improve individual foraging success, especially when food is scarce and distributed heterogeneously^72^. It is also possible that larval aggregation alters the nutritional composition and the microbial communities of the diets. For instance, larvae of some insect species can be cannibalistic^73,74^, and because larvae are a rich source of nutrients, cannibalism could affect the nutrient status of a food patch. Moreover, in *D. melanogaster*, larval foraging behaviour is determined by the bacterial communities in the diet^75^, and in *B. tryoni*, gut-microbial fungi in the diet have been found to promote larval development under nutrient-limiting conditions^76^. If larval density affected the relative abundance of these fungi in the diet, this could in turn have influenced larval foraging behaviour and larval body mass. Future studies that investigate the impact of larval density on the occurrence of cannibalism, and that compare changes in larval and diet microbial profiles in high- and low-density social environments will provide insights into the mechanisms underpinning the effects of larval environments on foraging behaviour and growth.

A negative relationship between population density and individual fitness is often assumed in ecology [reviewed by^77^]. In invertebrates, including tephritid fruit flies, high-densities at the larval stage can decrease nutrient availability, and reduce adult body mass, reproductive success, and survival [e.g.^1,3–9,60^], which can lead to a density-dependent effects on fitness that extends through generations^6^. However, high densities can also mitigate the negative effects of environmental stresses, and act as a buffering factor for individual fitness and survival [reviewed by^77^]. Therefore, high-density environments can sometimes confer fitness benefits. Our findings support this view, as they reveal that the density of larvae can trigger behavioural responses early in life that can benefit larval growth. This positive effect is likely due to an increase in exploratory behaviour when at high-densities, which can increase niche exploration and nutrient acquisition. It is important to mention that competition amongst conspecifics should determine threshold in which sociality provides benefits to the larvae, after which further increase in density should incur costs that offset the benefits to individuals’ fitness^45^. This threshold is currently unknown, but we predict that further increase in the density of larvae in our experiments (e.g., 400 larvae) should result in measurable costs such as decrease in body mass of the larvae. Determining the threshold is out of the scope of this study but remains an important topic for future investigations. Nonetheless, our findings are applicable to biological scenarios where intraspecific competition increases and resources are heterogeneous, and thus represent a logical consequence of the interaction between the nutritional and social environments.

It is important to mention that as density increases, larvae may be displaced from the patch due to the competition with conspecifics for space. This is a natural consequence of high larval density (i.e., defined as more larvae per unit of space), and understanding how the competition for space underlies larval behaviour is an important topic for future investigations. Also, patch quality could have decreased over time, especially in treatments with high larval densities, and influenced some of the results found in our study. This is unlikely, however, because the number of individuals in each patch sharply increased and stabilised in a plateau, with no evidence of larvae evasion from the chosen patches throughout the 24 h in which the experiment was conducted (see e.g., Fig. S3). Thus, our results demonstrate how the interactions between larval density and larval nutritional environment shape larval foraging behaviour.

## Conclusion

The present study provides a new perspective on density-dependent effects on larval development. Fruit fly larvae respond to a range of social and nutritional factors, with important implications for larval foraging and growth. Together, our findings help us understand the ecological factors underpinning larval development in insects, and serve as an important stepping-stone for future research aimed at better understanding the behavioural and nutritional aspects of development in group-living insects.

### Data Accessibility

The data is available in Dryad: 10.5061/dryad.b8p41t8.

## Electronic supplementary material

Supplementary Information
