# Supplementary material for: Social and nutritional factors shape larval aggregation, foraging, and body mass in a polyphagous fly
Source: Sci Rep. 2018 Oct 3;8:14750. doi: 10.1038/s41598-018-32930-0 (PMC6170467; doi:10.1038/s41598-018-32930-0)
Supplement: Supplementary file 1 — Supplementary Information [file 41598_2018_32930_MOESM1_ESM.docx]

**Supplementary Information: “Social and nutritional factors shape larval aggregation, foraging, and body mass in a polyphagous fly”**

Authors: Juliano Morimoto^1,2,*^, Binh Nguyen^1^, Shabnam Tarahi Tabrizi^1^, Fleur Ponton^1^, Phillip Taylor^1^

1 - Department of Biological Sciences, Macquarie University, NSW 2109, Australia

2 - Programa de Pós-Graduação em Ecologia e Conservação, Federal University of Paraná, Curitiba, Brazil, 19031, CEP: 81531-990

* To whom correspondence should be addressed.

E-mail: [juliano.morimoto@mq.edu.au](mailto:juliano.morimoto@mq.edu.au)

**Figures**

**Figure S1 - Design of the foraging arenas.** (a) Schematic representations of the foraging arena used in our larval aggregation experiments. Note that the arenas were designed exactly as in (b), although all patches contained the same diet concentration. (b) Schematic representations of the foraging arena used in our larval dietary choice experiments.

**Figure S2 – Box plots showing the behaviour of the residuals from the density-dependent simulation.** Note that we extrapolated our simulations to include foraging groups with density of 200 larvae (see Methods). Line was drawn using the ‘loess’ method in the package ‘ggplot2’ in R to highlight the trend in the data.

**Figure S3** – **Larval foraging behaviour.** The number of larvae in each foraging patch over time across the larval density treatments.

**Tables**

**Table S1 – Complete analysis of larvae body mass.** **Bold:** p <0.05

| **Response variable:** | **Larval body mass** | |  |  |  |  |
| --- | --- | --- | --- | --- | --- | --- |
| **Factors** | **Df** | **Sum Sq** | **Mean Sq** | **F-value** | **p-value** |  |
| Replicate | 1 | 0.193 | 0.193 | 0.1505 | 0.6994 |  |
| Diet | 4 | 151.032 | 37.758 | 29.4957 | **<0.001** | *** |
| Larval density | 3 | 223.345 | 74.448 | 58.1575 | **<0.001** | *** |
| Diet*Larval density | 12 | 16.698 | 1.392 | 1.087 | 0.3876 |  |
| Residuals | 59 | 75.527 | 1.28 |  |  |  |

**Table S2 – Complete analysis of larvae aggregation index.** Data was transformed (square-rooted) for statistical testing. **Bold:** p <0.05

| **Response variable:** | **(Agggregation index^2)^0.25** | | |  |  |  |
| --- | --- | --- | --- | --- | --- | --- |
| **Factors** | **Df** | **Sum Sq** | **Mean Sq** | **F-value** | **p-value** |  |
| Larval density | 3 | 0.2324 | 0.07746 | 2.2297 | 0.084 | . |
| Diet | 4 | 0.5862 | 0.14655 | 4.2183 | **0.002** | ** |
| Time | 1 | 0.0108 | 0.01085 | 0.3122 | 0.577 |  |
| Larval density*Diet | 12 | 1.1053 | 0.09211 | 2.6512 | **0.001** | ** |
| Larval density*Time | 3 | 1.1645 | 0.38815 | 11.1727 | **<0.001** | *** |
| Diet*Time | 4 | 0.0861 | 0.02151 | 0.6193 | 0.648 |  |
| Residuals | 452 | 15.7029 | 0.03474 |  |  |  |
| **Levene's Test** | **df** | **F-value** | **p-value** |  |  |  |
| Larval density | 3 | 1.329 | 0.264 |  |  |  |
|  | 476 |  |  |  |  |  |
| Diet | 4 | 0.234 | 0.918 |  |  |  |
|  | 475 |  |  |  |  |  |

**Table S3 – Complete analysis of the relationship between larvae body mass and aggregation index.** **Bold:** p <0.05

| **Response variable:** | **Larval body mass** | |  |  |  |  |
| --- | --- | --- | --- | --- | --- | --- |
| **Factors** | **Df** | **Sum Sq** | **Mean Sq** | **F-value** | **p-value** |  |
| Aggregation index | 1 | 0.05314 | 0.05314 | 12.4732 | **<0.001** | *** |
| Larval density | 1 | 0.75962 | 0.75962 | 178.3096 | **<0.001** | *** |
| Diet | 4 | 0.56166 | 0.14042 | 32.9603 | **<0.001** | *** |
| Aggregation index*Larval density | 1 | 0.00004 | 0.00004 | 0.0099 | 0.921 |  |
| Aggregation index*Diet | 4 | 0.01165 | 0.00291 | 0.6835 | 0.606 |  |
| Larval density*Diet | 4 | 0.04238 | 0.01059 | 2.4868 | 0.052 | . |
| Residuals | 64 | 0.27265 | 0.00426 |  |  |  |

**Table S4 – Complete analysis of the proportion of larvae in the most numerous aggregate.** GLM with Binomial distribution and quasi extension to account for overdispersion of the data.

| **Response variable:** | **Proportion of larvae in the biggest aggregate** | | | |  |  |  |
| --- | --- | --- | --- | --- | --- | --- | --- |
| **Factors** | **Df** | **Deviance** | **Residual Df** | **Residual Dev** | **F-value** | **p-value** |  |
| NULL |  |  | 479 | 47.173 |  |  |  |
| Time | 1 | 0.1264 | 478 | 47.046 | 1.5949 | 0.207 |  |
| Larval density | 3 | 4.4994 | 475 | 42.547 | 18.9285 | **<0.001** | *** |
| Diet | 4 | 0.7999 | 471 | 41.747 | 2.5237 | **0.040** | * |
| Time* Larval density | 3 | 1.8236 | 468 | 39.923 | 7.6717 | **<0.001** | *** |
| Time * Diet | 4 | 0.3005 | 464 | 39.623 | 0.9481 | 0.436 |  |
| Larval density * Diet | 12 | 2.236 | 452 | 37.387 | 2.3517 | **0.006** | ** |

**Table S5 - Complete analysis of larvae willingness to forage.** Agar (no choice) is the reference level. **Bold:** p <0.05

| **Food Patch** | **(Intercept)** | **SE** | **Patch order** | **SE** | **Density 25 larvae** | **SE** | **p-value** | **Density 50 larvae** | **SE** | **p-value** | **Density 100 larvae** | **SE** | **p-value** |
| --- | --- | --- | --- | --- | --- | --- | --- | --- | --- | --- | --- | --- | --- |
| *100%* | -0.449 | 0.387 | -0.293 | 0.106 | -0.960 | 0.494 | 0.052 | 0.037 | 0.415 | 0.928 | 0.043 | 0.407 | 0.917 |
| *80%* | -1.000 | 0.391 | 0.200 | 0.089 | 0.145 | 0.441 | 0.743 | 0.171 | 0.418 | 0.682 | 1.057 | 0.404 | **0.009** |
| *60%* | -0.723 | 0.341 | 0.444 | 0.098 | -0.386 | 0.410 | 0.346 | -0.663 | 0.383 | 0.083 | 0.322 | 0.358 | 0.368 |
| *40%* | -0.901 | 0.439 | 0.438 | 0.110 | 0.487 | 0.486 | 0.317 | -0.350 | 0.471 | 0.458 | -0.221 | 0.459 | 0.630 |
| *20%* | -0.472 | 0.406 | 0.080 | 0.143 | -0.792 | 0.502 | 0.114 | -0.319 | 0.460 | 0.488 | -0.567 | 0.438 | 0.195 |
|  |  |  |  |  |  |  |  |  |  |  |  |  |  |
| **Food Patch** | **Time** | **SE** | **p-value** | **Time*Density 25 larvae** | **SE** | **p-value** | **Time*density 50 larvae** | **SE** | **p-value** | **Time* Density 100 larvae** | **SE** | **p-value** |  |
| *100%* | 0.105 | 0.090 | 0.244 | 0.012 | 0.114 | 0.917 | 0.144 | 0.098 | 0.142 | 0.069 | 0.096 | 0.474 |  |
| *80%* | 0.232 | 0.085 | **0.006** | 0.111 | 0.097 | 0.252 | 0.150 | 0.093 | 0.105 | 0.094 | 0.090 | 0.293 |  |
| *60%* | 0.313 | 0.075 | **0.000** | -0.114 | 0.091 | 0.213 | -0.031 | 0.086 | 0.719 | -0.067 | 0.081 | 0.407 |  |
| *40%* | -0.025 | 0.113 | 0.825 | 0.032 | 0.126 | 0.797 | 0.269 | 0.121 | **0.026** | 0.242 | 0.118 | **0.041** |  |
| *20%* | -0.017 | 0.103 | 0.865 | 0.030 | 0.128 | 0.815 | -0.157 | 0.129 | 0.224 | 0.026 | 0.114 | 0.819 |  |

**Table S6 - Complete analysis of larvae dietary choices.** Standard diet (100% macronutrient concentration) as reference level. *** non-overlapping 99% CI**.**

| **Larvae density**: 10 larvae |  |  |  |  |  |
| --- | --- | --- | --- | --- | --- |
|  | Relative | Log-odds | 99% CI time | |  |
| Patch (relative to 100%) | Time | SE | lwr | upr |  |
| 80% | 0.1289399 | 0.08684132 | -0.09474853 | 0.3526283 |  |
| 60% | 0.2108781 | 0.07667436 | 0.01337803 | 0.4083782 | *** |
| 40% | -0.1287073 | 0.1155807 | -0.4264235 | 0.1690088 |  |
| 20% | -0.1336627 | 0.10595722 | -0.4065904 | 0.139265 |  |
| **Larvae density**: 25 larvae |  |  |  |  |  |
|  | Relative | Log-odds | 99% CI time | |  |
| Patch (relative to 100%) | Time | SE | lwr | upr |  |
| 80% | 0.22921785 | 0.06616445 | 0.05878953 | 0.3996462 | *** |
| 60% | 0.08479123 | 0.07091934 | -0.09788488 | 0.2674673 |  |
| 40% | -0.12493808 | 0.07444799 | -0.3167034 | 0.06682722 |  |
| 20% | -0.11307588 | 0.09135244 | -0.3483842 | 0.1222324 |  |
| **Larvae density**: 50 larvae |  |  |  |  |  |
|  | Relative | Log-odds | 99% CI time | |  |
| Patch (relative to 100%) | Time | SE | lwr | upr |  |
| 80% | 0.149129763 | 0.03021597 | 0.07129859 | 0.2269609 | *** |
| 60% | 0.040826922 | 0.03679049 | -0.0539391 | 0.1355929 |  |
| 40% | 0.000994144 | 0.03728374 | -0.0950424 | 0.09703069 |  |
| 20% | -0.448329829 | 0.07889534 | -0.6515508 | -0.2451089 | *** |
| **Larvae density**: 100 larvae |  |  |  |  |  |
|  | Relative | Log-odds | 99% CI time | |  |
| Patch (relative to 100%) | Time | SE | lwr | upr |  |
| 80% | 0.15492918 | 0.02483084 | 0.09096917 | 0.2188892 | *** |
| 60% | 0.07368724 | 0.02726425 | 0.003459185 | 0.1439153 |  |
| 40% | 0.04386088 | 0.03269745 | -0.040362183 | 0.1280839 |  |
| 20% | -0.16891233 | 0.04822348 | -0.2931278 | -0.04469687 | *** |

**Table S7 - Diet recipes –** The formulations for the diets used in the experiments.

| **Ingredient** | **100%** | **80%** | **60%** | **40%** | **20%** |
| --- | --- | --- | --- | --- | --- |
| **Hydrolyzed Yeast (g)** | 20.4 | 16.32 | 12.24 | 8.16 | 4.08 |
| **Sugar (g)** | 12.18 | 9.74 | 7.3 | 4.87 | 2.43 |
| **Agar(g)** | 1 | 1 | 1 | 1 | 1 |
| **Citric Acid (g)** | 2.3 | 2.3 | 2.3 | 2.3 | 2.3 |
| **Nipagen (g)** | 0.2 | 0.2 | 0.2 | 0.2 | 0.2 |
| **Sodium benzoate (g)** | 0.2 | 0.2 | 0.2 | 0.2 | 0.2 |
| **Wheat Germ Oil (ml)** | 0.2 | 0.2 | 0.2 | 0.2 | 0.2 |
| **MiliQ Water (ml)** | 100 | 100 | 100 | 100 | 100 |
